# The Adaptive Change of HLA-DRB1 Allele Frequencies Caused by Natural Selection in a Mongolian Population That Migrated to the South of China

**DOI:** 10.1371/journal.pone.0134334

**Published:** 2015-07-31

**Authors:** Hao Sun, Zhaoqing Yang, Keqin Lin, Shuyuan Liu, Kai Huang, Xiuyun Wang, Jiayou Chu, Xiaoqin Huang

**Affiliations:** Institute of Medical Biology, Chinese Academy of Medical Sciences and Peking Union Medical College, Kunming 650118, China; Universitat Pompeu Fabra, SPAIN

## Abstract

Pathogen-driven balancing selection determines the richness of human leukocyte antigen (HLA) alleles. Changes in the pathogen spectrum may cause corresponding changes in HLA loci. Approximately 700 years ago, a Mongolian population moved from the north of China to the Yunnan region in the south of China. The pathogen spectrum in the south of China differs from that in the north. In this study, changes in the HLA genes in the Yunnan Mongolian population, as well as the underlying mechanism, were investigated. A sequence-based typing method (SBT) was used to genotype HLA-DRB1 in 470 individuals from two Mongolian populations and another five ethnic groups. Meanwhile, 10 autosomal short tandem repeats (STRs) were genotyped to assess the influence of genetic background on HLA-DRB1 frequencies. The frequencies of certain alleles changed significantly in the Mongolian population that migrated to Yunnan. For example, DRB1*12:02:01 increased from 6.1% to 35.4%. STR analysis excluded the possibility of a recent bottleneck and indicated that 50% of the genetic consistency between northern and southern Mongolians; Tajima's *D* value for HLA-DRB1 exon2 and *d*N*/d*S analysis showed that the HLA-DRB1 genes in both Mongolian populations were under balancing selection. However, the sites under natural selection changed. We proposed that the dramatically change of HLA frequencies in southern Mongolian was caused by a combination of inter-population gene flow and natural selection. Certain diseases specific to the south of China, such as malaria, may be the driving force behind the enhanced DRB1*12:02:01 frequency.

## Introduction

In the human genome, the human leukocyte antigen (HLA) gene is thought to be the most polymorphic. HLA class I has more than 8,000 alleles, and HLA class II has nearly 2,000 alleles, of which HLA-DRB1 has thus far been found to have at least 1,400 alleles, according to the statistics of the IMGT/HLA database in 2013.[1] The main function of the proteins encoded by the HLA class I or class II alleles is to present intracellular or exogenous antigen peptides to CD8 ^+^or CD4 ^+^ T cells and trigger downstream immune responses,[2] thus playing an important role in defending against pathogen invasion. Many scientists believe that pathogen-mediated selection (PMS) has contributed to the highly polymorphic nature of the HLA genes.[3, 4] Therefore, as the pathogen challenge changes, the frequency of the corresponding HLA alleles in a population fluctuates according to the changing selective pressure.

Different types and intensities of pathogen (pathogen spectrum) exist in southern and northern China. Tropical southern China is hot and humid, whereas northern China is cold and dry; different climate conditions lead to the prevalence of different types of pathogens.[5, 6] Particularly in Yunnan Province in southern China, a variety of pathogens are notably active.[7] Therefore, when a population migrates from the north to the south, the HLA genes of the population should adapt to the altered pathogen spectrum. Around the year 1250, under the command of Kublai Khan, a military legion of approximately 100,000 Mongol troops entered southern China from the northern temperate grasslands, which were then part of the legion settled in Tonghai Prefecture of Yunnan Province, thus forming the Mongolian population in Yunnan. In this study, we compare the HLA allele frequencies between the Mongolian population in Yunnan (Mongolian_YN) and the Mongolian population in Inner Mongolia (Mongolian_IM) to investigate whether the changes in selective pressure brought about by the changed pathogen spectrum drove changes in the distribution of HLA allele frequencies in Mongolian_YN.

Because the main role of HLA class II alleles is to present exogenous antigen peptides and because subclass HLA-DRB1 alleles are the most abundant of the class II genes, HLA-DRB1 alleles were targeted in this study. In addition to the selective pressure derived from environmental factors, demographic events such as genetic drift caused by bottleneck effects also affect MHC polymorphisms.[8] Therefore, we also analyzed 10 neutral short tandem repeats (STRs) in both populations to assess the impact of demographic events on changes in HLA frequency. Yunnan Province is populated by many ethnic minority groups; thus, genetic exchange may have occurred between the indigenous population and Mongolian_YN after migration. For this reason, according to the genetic background of the Yunnan population distribution[9, 10], four ethnic minority groups native to Yunnan for hundreds of years, i.e., the Wa people, the Hani people, the Dai people, and the Yao people, were also analyzed in addition to Mongolian_YN and Mongolian_IM. Furthermore, the Han people, which dominates the composition of the population in China, was also included in the analysis. In total, 470 individuals from seven populations were investigated.

## Materials and Methods

### Sampled populations

**Fig 1:**
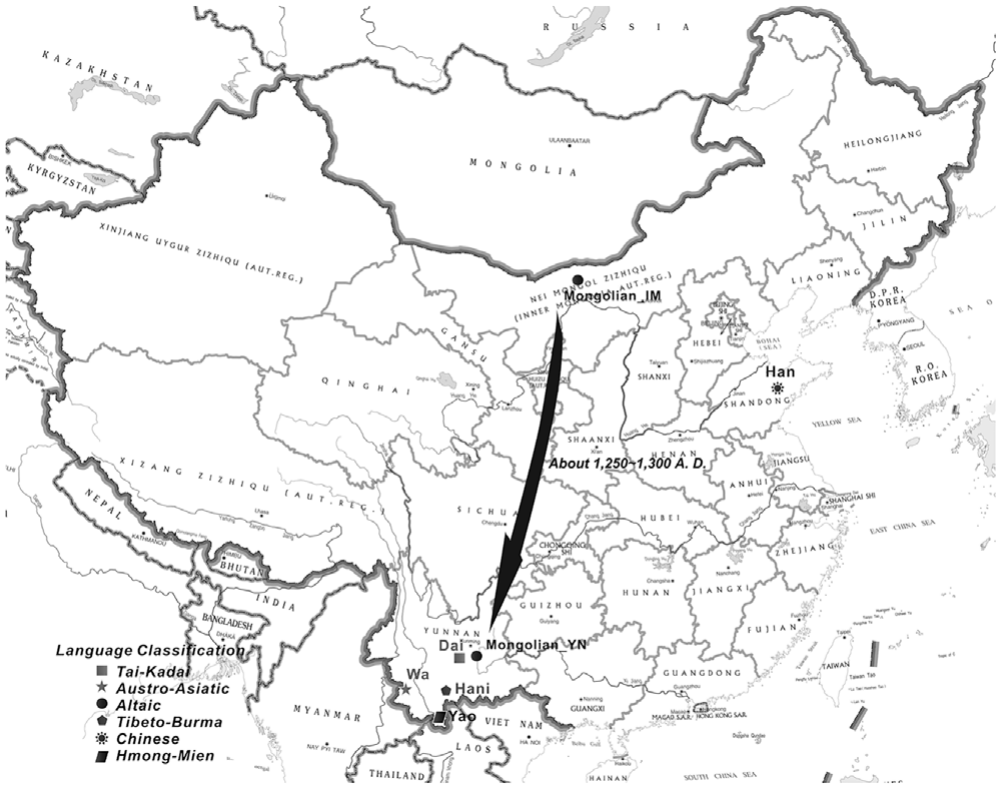
Information on the seven sampled populations. Geographical location and language classification of the seven sampled populations.

To study changes in HLA-DRB1 allele polymorphism after Mongolian migration from the north to the south, the HLA-DRB1 alleles from 49 individuals of Mongolian descent from Inner Mongolia (Mongolian_IM) and from 103 individuals of Mongolian descent from Yunnan Province (Mongolian_YN) were genotyped. Meanwhile, the alleles of individuals from another four ethnic minority groups (Wa, Hani, Dai, and Yao) and one ethnic majority group (Han) were also genotyped to address the effect of gene exchange among the populations on HLA-DRB1allele frequencies. Thus, the samples included 470 individuals from seven populations. Sample information and the place of residence of the subjects are shown in Table 1 and Fig 1. Mongolian_IM and the Han people were from northern China, whereas the other populations were from southern China. Furthermore, 48 individuals were randomly selected from the 103 Mongolian_YN and subjected to genotyping of 10 neutral STRs of autosomal origin. STR data for the remaining six populations in this study were derived from our previous work.[9, 10] These data were also used to address demographic events, such as the impact of the bottleneck effect on the population.

**Table 1:** Information about the seven populations sampled for HLA-DRB1 genotyping.

No.	Population	Size	Location	Lat(N)	Long(E)	Language classification
1	Han	49	Zouping, Shandong	36.86	117.74	Chinese
2	Mongolian_IM	49	Damaoqi, Inner Mongolia	41.70	110.43	Altaic, Mongolic
3	Mongolian_YN*	103	Tonghai, Yunnan	24.15	102.67	Altaic, Mongolic
4	Hani	67	Jinghong, Yunnan	22.01	100.79	Tibeto-Burman
5	Dai	65	Xinping, Yunnan	24.07	101.99	Tai-Kadai
6	Yao	60	Mengla, Yunnan	21.48	101.57	Hmong-Mien
7	Wa	77	Ximeng, Yunnan	22.64	99.60	Austro-Asiatic
	Total	470				

Lat and Long represent latitude (north) and longitude (east), respectively.

* 10 STR analyses were performed from 48 members of this population.

### Ethics statement and DNA preparation

Blood samples were collected and transformed into cell lines in the Chinese Human Genome Diversity Project.[11] Written informed consent was obtained from each donor for the use of these cells or blood samples in research. The Ethics Committee of the Chinese Academy of Medical Sciences and Peking Union Medical College approved this project. All of the DNA samples were extracted from immortalized cell lines using a DNA Miniprep Kit (Axygen, China).

### HLA-DRB1 and STR genotyping

Changes in antigen recognition sites may best reflect the relationship between selective pressure and gene mutations. Because the antigen recognition site is encoded by HLA-DRB1exon 2, this region was selected as the target fragment for genotyping via sequence-based typing (SBT) based on the method of D. Sayer et al.[12], in which HLA-DRB1 genotyping was performed by sequencing the product of a single PCR run. In this study, two PCR runs were performed for each sample, and four sequencing runs were performed for each PCR product to achieve a more accurate HLA-DRB1genotype. For the two PCR runs, the same mixture of eight primers (DRB1-52, DRB1-52.1, DRB1-01, DRB1-07, DRB1-09, DRB1-10, DRB1-15, and DRB1-04)[12] at equal molarity was used as the forward primer. The reverse primers for the two PCR runs were different; DRB1-87.M13F was used in one run, and DRB1-91.M13F was used for the other.[12] The PCR reactions were performed in a total volume of 50 μl, which consisted of genomic DNA (50 ng), forward primer mixture (25 pmol of each primer), reverse primer (50 pmol), Transtart DNA polymerase (1.25U; Transgen, China), 10nmol of each deoxynucleotide triphosphate (dNTP) (Transgen, China), and 1× PCR Buffer (Transgen, China). PCR amplification was performed in a Perkin Elmer Gene Amp PCR System 9700 thermal cycler (Applied Biosystems, USA), and the cycling conditions were as follows: initial denaturation at 94°C for 5 min; 35 cycles of 94°C for 20 s, 63°C for 10 s, and 72°C for 90 s; and a final extension at 72°C for 15 min. The PCR products were sequenced using M13F Big Dye Primer Ready Reaction kits (Applied Biosystems, USA) and an automated 3730xl DNA Analyzer (Applied Biosystems, USA). The results of the two sequencing runs were aligned using the SBTengine software package (GENDX, Netherlands) to visualize the genotypic composition of the HLA-DRB1 alleles. Based on the predicted allele, an allele-specific primer was used to sequence the longer PCR product produced by the PCR reaction with the forward primer mixture and DRB1-91.M13F. The allele-specific primers[12] included seven primers, i.e., DRB1-52, DRB1-01, DRB1-07, DRB1-09, DRB1-10, DRB1-15, DRB1-04, and the group-specific sequence primers (GSSPs) proposed by the SBTengine software (GENDX, Netherlands). For example, for the pre-genotyping result of HLA-DRB1*15:02:01/12:02:01, DRB1-15 and DRB1-52 were used to sequence the longer PCR products. If the pre-genotyping result showed that the allele-specific primer could not distinguish the genotypes, e.g., HLA-DRB1*12:02:01/03:01, then the GSSP primer was used for sequencing. The results from the four sequencing runs were aligned using SBTengine software to achieve the best genotyping result.

The 10 STRs were used to study the genetic backgrounds of the populations. In this study, 10 STRsfrom48 individuals who were randomly selected from the 103 Mongolian_YN individuals were genotyped. The information on these sites and the genotyping method were the same as previously reported.[10] The CEPH 1347–02 standard (Applied Biosystems, USA) was genotyped to ensure that the genotyping conditions were consistent with those reported previously. The STR genotyping data of the remaining six populations were derived from our previous work.[9, 10]

### Genetic diversity tests

The GenAlEx 6.4 package[13] was used to analyze the gene frequencies of STRs and HLA alleles and to convert the data format. The observed heterozygosities (*Ho*), expected heterozygosities (*He*), and fixation index F (F = (*He*—*Ho*) / *He*) of HLA-DRB1 and the 10 STRs of the seven populations were also calculated using the same software package. The loci were tested for Hardy-Weinberg equilibrium using Arlequin 3.5 software.[14] The gene frequencies of HLA-DRB1 alleles between the different populations were compared using the R × C chi-squared test and 2 × 2 chi-squared test.

### Neutral equilibrium tests

A variety of methods can be used to test the neutral-equilibrium model and investigate the effect of selection on target genes.[15] In this study, two methods that are based on sequence data were employed to analyze the selection effects on HLA-DRB1 in different populations. The first method was to analyze the frequencies at which polymorphisms occur in a series of DNA sequences collected from a population, the site frequency spectrum of the sequences, to deduce whether selection has occurred.[15, 16] The DnaSP V5 software[17] was used for the test that is based on the site frequency spectrum: Tajima's *D* test[16]. The significance of Tajima’s D was also computed by coalescent simulation in this software. The widely used Tajima's *D* test is based on the comparison of two measures of the neutral parameter θ. The parameter θ can be estimated by the mean number of differences among DNA sequences (mean pairwise difference, π) and can also be estimated based on the number of polymorphic sites (θs). Under purifying selection (i.e., negative selection), most novel variants reduce the fitness of the individual carriers and rarely rise to high frequencies. Thus, there is an excess of low-frequency variants, resulting in a higher θs and a Tajima’s *D*< 0. Under balancing selection, selection favors the maintenance of different alleles in the population and increases the proportion of variants at intermediate frequencies. This will result in a proportionally higher mean pairwise difference (π) compared with the measure of diversity based on the number of polymorphic sites (θs) and thus Tajima’s *D*> 0.

The second method, discerning selection, is a based on differences between nonsynonymous and synonymous substitution rates. Nonsynonymous mutations are much more likely than synonymous changes to have an effect on fitness. Thus, the ratio (*ω* = *d*_N_/*d*_S_) of the number of nonsynonymous substitutions per nonsynonymous site (*d*_N_) and the number of synonymous substitutions per synonymous site (*d*_S_) provides a sensitive measure of selective pressure at the protein level, with ω values < 1, = 1, and > 1 indicating purifying selection, neutral evolution, and positive selection, respectively.[15, 18] The likelihood ratio test (LRT) was used to compare the differences between nonsynonymous and synonymous substitution rates. This test was performed by using PAML 4[19] and PAMLX[20] software. The phylogenetic trees required for analysis were generated using MEGA6 via the neighbor-joining (NJ) method, in which the type of nucleic acid mutations was set using the default settings in the PAML software. Because the antigen recognition site (ARS) of HLA may best reflect the effects of selective pressure on the gene[21], the sequence of HLA-DRB1exon 2,which contains the ARS sequence (from codon 7 to 90), was used in these analyses.

### Detection of bottlenecks

For all seven populations, the recent occurrence of bottlenecks was analyzed using BOTTLENECK 1.2.02 software[22] based on the STR data. The BOTTLENECK program compares a sample's heterozygosity (*He*) at each locus with the expected heterozygosity under mutation-drift equilibrium (*Heq*) by the Wilcoxon test. Excess heterozygosity (*He> Heq*) suggests a population contraction (i.e., a bottleneck), whereas a heterozygosity deficit (*He< Heq*) suggests a population expansion.[23] We used a stepwise mutation model (SMM) and a two-phase model (TPM) with parameters recommended by the program's authors (non-stepwise = 5%, variance = 12) to perform these tests because these models may be more realistic for STR markers.[22]

### Population structure

To analyze the impact of demographic events on the population, the paired fixation index F (*F*_*ST*_) was calculated for the seven populations based on the 10 neutral STRs using the Poptree2 package.[24] To further investigate the genetic structure of populations based on information from the 10 STRs, a model-based clustering method in the STRUCTURE V2.3 program[25] was used to estimate the reasonable partitions of these populations. The location of samples (LOCPRIOR model[26]) was used to detect weak population structures. The other STRUCTURE parameters were established according to the suggestions of Falush et al.[27] The degree of admixture alpha was inferred from the data; the admixture model was chosen, and the correlations of allele frequencies between populations were taken into account. Population structures were inferred by setting the value of the clusters (*K*) from 2 to 9. Ten runs were performed for every K value, with an MCMC chain burn-in length of 80,000 iterations followed by 80,000 iterations. True K was identified using the value of the average logarithmic probability across runs returned by STRUCTURE 2.3 and the method of Evanno et al.[28] These identifications were implemented by the online software STRUCTURE HARVESTER.[29] Outputs from STRUCTURE were graphically modified by DISTRUCT.[30]

### Gene flow

For the populations with possible genetic exchange with Mongolian_YN, gene flow was simulated based on the sequence data of HLA-DRB1 using DIYABC 2.0 software.[31] For each possible case of genetic exchange (later referred to as a scenario), 500,000 simulations were performed. The "evaluate scenario-prior combination" option in the software was used to calculate the summary statistics for the simulated data sets and the real (observed) data set. The summary statistics included 13 population genetics parameters such as number of haplotypes, number of segregating sites, mean of pairwise differences, etc. A principal component analysis (PCA) was performed in the space of summary statistics on 10,000 simulated data sets, and the target (observed) data set was added on each plane of the analysis to evaluate how the latter was surrounded by the simulated data sets. If the observed data are not surrounded by simulated data sets, then proven scenarios are not well established to interpret the real situation.

When setting up the scenario, to better approximate the real situation, unknown demographic parameters were established as a widely variable range. Effective population sizes (Ne) of all populations were set from10 to 30,000 because the Ne of the general human population is approximately 10,000.[32] According to historical records, the Mongols immigrated to Yunnan 750 years ago, thus separating the north and south of the Mongolian population at time t1,which equates to28 to 32 generations (assuming 25 years per generation). The separation time of other the populations is 10 to 2,400 generations because the earliest humans entered East Asia approximately 60,000 years ago[33], and these populations should only have separated thereafter. Admixture proportions were set in the range of 0.001 to 0.999. The individual locus mutation rate was set to 1.00E-9 to 1.00E-6, which was in line with the gamma distribution.

DIYABC 2.0 software was also used to infer the most possible scenario for Mongolian_YN based on the STR data. The demographic parameters were the same as the HLA-DRB1 analysis except the mutation rate was set to 1.00E-4 to 1.00E-3.

## Results

### Genetic diversity tests

The HLA alleles from a total of 470 individuals from seven populations were successfully genotyped; the allele frequencies and polymorphism statistics are shown in S1 Table. Ambiguous alleles are listed in the table notes. Ambiguous alleles with the same sequence as HLA-DRB1 exon 2 were considered a variant in the selection analyses because they experienced the same selective pressure. For the 48 individuals from Mongolian_IM, genotyping based on the 10 STRs was also successfully conducted, and the results are listed in S2 Table, including the results for the six populations from our previous work. In all seven populations, the distributions of the genotype frequencies of the HLA alleles were consistent with Hardy-Weinberg equilibrium. Additionally, HLA-DRB1 displayed a high degree of polymorphism in all of the populations, with *He* ranging from0.774–0.935. Except for the Han people, the *Ho* of each of the other six populations was larger than the expected heterozygosity *He*, i.e., *F* was negative. After Bonferroni correction, the genotype frequencies of the 10 STRs in the Mongolian_YN population also displayed Hardy-Weinberg equilibrium and exhibited a high rate of polymorphism. The distribution of *F* values for each population and each locus assumed a random distribution.

### Detection of bottlenecks

Both the expansion of a population and the bottleneck effect can alter the distribution of allele frequencies. In particular, the bottleneck effect may have a similar outcome to that of balancing selection, leading to an increased frequency of alleles with moderate frequencies.[15, 34] Therefore, based on the STR data, recent occurrences of bottlenecks were analyzed in the seven populations using the BOTTLENECK software, and the results are shown in Table 2. Regardless of the mode used (SMM or TPM), excess heterozygosity (*He*>*Heq*) was not observed. The allele frequency distributions for the seven populations were all L-shaped. At mutation-drift equilibrium, the rarest allele class is expected to be much more frequent than the second-rarest class, so the allele frequency distribution is L-shaped. However, because the rarest alleles are rapidly lost after a bottleneck, this category of allele proportions drops, and the characteristic L-shaped distribution of allele proportions no longer exists. Therefore, the above analyses suggest that there have been no bottleneck effects in the recent history of the seven populations.

**Table 2:** Results from the BOTTLENECK tests using 10 microsatellites for seven populations.

Population(n)	Wilcoxon test P values in TPM	Wilcoxon test P values in SMM	Allele frequency distribution
Han (n = 90)	0.920	0.984	L-shaped
Mongolian_IM (n = 100)	0.813	0.991	L-shaped
Mongolian_YN (n = 96)	0.461	0.688	L-shaped
Hani (n = 110)	0.997	0.999	L-shaped
Dai (n = 120)	0.754	0.935	L-shaped
Yao (n = 102)	0.423	0.615	L-shaped
Wa (n = 98)	0.577	0.722	L-shaped

n represents the number of chromosomes; TPM, SMM, and L-shaped are defined in the text; Wilcoxon test P values are one-tailed tests of excess heterozygosity (*H*_e_>*H*_eq_).

### Neutral equilibrium tests

The results of Tajima's *D* test for the HLA-DRB1exon 2 sequence data are listed in Table 3. In our results, Tajima's *D* was significantly greater than zero for all populations. Therefore, when the site frequency spectrum was used to test selection, all the populations were found to be under balancing selection.

**Table 3:** Test of the neutrality-equilibrium model based on the site frequency spectrum at exon 2 of HLA-DRB1 in seven populations of China.

Population(n)	Tajima's D	P
Han(n = 98)	3.32	P = 0.0002
Mongolian_IM(n = 98)	2.96	P = 0.0005
Mongolian_YN(n = 206)	2.93	P = 0.0015
Hani(n = 134)	2.52	P = 0.0053
Dai(n = 130)	2.63	P = 0.0035
Yao(n = 120)	2.21	P = 0.0120
Wa(n = 154)	2.82	P = 0.0035

Positive selection often acts on a few sites, and the signal may be swamped by ubiquitous negative selection. Yang and Nielsen (2002) introduced a site model for testing positive selection on individual codons. Based on the above method, the selection on the codons of exon 2 of HLA-DRB1 in the seven populations was analyzed using the PAML program. The results from Mongolian_IM and Mongolian_YN are shown in Table 4, and the results from the remaining five populations are shown in S3 Table.

**Table 4:** Log-likelihood values and parameter estimates for HLA-DRB1 exon 2 in Mongolian_YN and Mongolian_IM.

Pop	Model code	λ	Average *d*_N_/*d*_S_	Parameter estimates*
Mongolian_YN	**M1a**(nearly neutral)	-1248.0	0.295	*p*0 = 0.719, *p*1 = 0.281; *ω*0 = 0.019, *ω*1 = 1.000
	**M2a**(positive selection)	-1224.1	0.708	*p*0 = 0.689, *p*1 = 0.198, *p*2 = 0.113; *ω*0 = 0.022, *ω*1 = 1.000, *ω*2 = 4.387
	**M7**(beta)	-1249.7	0.218	a = 0.014, b = 0.045
	**M8**(beta&*ω*>1)	-1226.1	0.708	*p*0 = 0.880, *p*2 = 0.120; a = 0.019, b = 0.062, *ω*2 = 4.300
Mongolian_IM	**M1a**(nearly neutral)	-1163.7	0.301	*p*0 = 0.714, *p*1 = 0.286; *ω*0 = 0.020,*ω*1 = 1.000
	**M2a**(positive selection)	-1143.4	0.730	*p*0 = 0.682, *p*1 = 0.198, *p*2 = 0.120 *ω*0 = 0.023, *ω*1 = 1.000, *ω*2 = 4.311
	**M7**(beta)	-1164.3	0.316	a = 0.013, b = 0.025
	**M8**(beta&*ω*>1)	-1144.1	0.713	*p*0 = 0.875, *p*2 = 0.125; a = 0.052, b = 0.182, *ω*2 = 4.146

*ω0 is theratio of nonsynonymous-synonymous substitutions (*d*_N_/*d*_S_) of sites, with the proportion of *p*0, at which nonsynonymous mutations are “slightly deleterious”; *ω*1 is the *d*_N_/*d*_S_ of completely neutral sites (*ω*1 = 1) with a proportion of *p*1; *ω*2 is the *d*_N_/*d*_S_ of positively selected sites with a proportion of *p*2. The ‘a’ and ‘b’ are the shape parameters of the beta distribution.

In the PAML program, the two-pair mutation models were used for the analysis.[35] One pair is the null model M1a (nearly neutral), which assumes two site classes with proportions *p*0 and *p*1 = 1—*p*0, with 0 <ω0 < 1 and ω1 = 1, and the alternative model M2a (positive selection), which adds a proportion *p*2 for sites with ω2> 1 estimated from the data. The other pair is M7 (beta), which assumes a beta distribution for ω (in the interval 0 <ω< 1), and the alternative model M8 (beta&ω), which adds an extra class of sites with positive selection (ω2>1). In Table 4 and S3 Table, the average ω of exon 2 of the seven populations in all models was between 0.2 and 0.7, indicating the dominant role of purifying selection in the evolution of HLA-DRB1. However, for all populations, the log likelihood values using models that allow for the existence of positive selection (M2a or M8) were significantly greater than those of the models that do not allow for the existence of positive selection (M1a or M7). For example, in the Mongolian_YN population, the LRT statistic for the comparison of M2a and M1a is 2Δ*l* = 2 × [-1224.1-(-1248.0)] = 47.8, and the null model (M1a) is rejected with a marginal P = 4 × 10^−11^ with d.f. = 2. The LRT statistic for the comparison of M8 and M7 is 2Δ*l* = 47.2, which is also much greater than the critical values from a χ2 distribution with d.f. = 2. This result showed that some codons in HLA-DRB1 exon 2 were all affected by selection in the seven populations.

In Mongolian_YN and Mongolian_IM, the proportions of sites under selective pressure were similar. In the two populations, approximately 70% (*p*0) of the sites were under strong purifying selection (ω0 was approximately 0.02), whereas approximately12% of sites were under strong positive selection (ω was 4.1–4.4). These results demonstrate that diversified selection has acted on HLA-DRB1 in the two populations, and the percentage of sites that were under positive selection was not significantly different between the two populations. In the other five populations, the proportions of sites under positive selection were approximately2.4% to 11.8%.

The sites under positive selection were predicted by using Bayes Empirical Bayes analysis (EBE)[35] in PAML software. The results are shown in Table 5. Different sites were under positive selection in the two Mongolian populations, although the proportions of sites under positive selection were similar. The 57^th^ amino acid was positively selected in Mongolian_YN but not in Mongolian_IM. On the other hand, the 74^th^ amino acid was positively selected in Mongolian_IM but not in Mongolian_YN. Residue 74 of HLA-DRB1 is within the antigen-binding groove of DR1. The aspartic acid residue β57 in DR1 forms a salt bridge with a conserved arginine α76.[36], indicating that these two sites, which under different selective pressure in the two Mongolian populations, can affect the protein function.

**Table 5:** Positively selected amino acid sites in HLA-DRB1 predicted by using Bayes Empirical Bayes analysis under the M2a model in seven populations.

Populations	Positively selected amino acid sites (Reference sequence P04229)								
	11	13	30	37	57	67	70	74	86
**Han**	**	**		**			**	**	**
**Mongolian_IM**	**	**				*	**	**	**
**Mongolian_YN**	**	**			**		**		**
**Dai**	*	**		*	**				**
**Wa**	*	**			**				**
**Yao**	**	**			**		*	*	
**Hani**		**							

** the probability of this site under positive selectiongreater than 0.99, Pr(ω> 1)> 0.99.

* Pr(ω> 1) > 0.95.

### Population structure

The genetic background of a population can help determine which action, selection or demographic events, has changed the gene frequency in the population. Therefore, the pairwise *F*_*ST*_ between the populations was calculated based on the 10 STRs and the HLA-DRB1 gene frequencies in the seven populations; the results are shown in S4 Table and S5 Table. *F*_*ST*_ values are shown in the lower triangle of the tables, and P values are shown in the upper triangle. All the pairwise *F*_*ST*_ values based on STR data are significantly different (α = 0.002 after Bonferroni correction), meaning that all paired populations have different genetic backgrounds. The *F*_*ST*_ between Mongolian_YN and Mongolian_IM was 0.015, which is smaller than the *F*_*ST*_ between Mongolian_YN and the other southern populations (0.018–0.026). Therefore, in terms of genetic background, Mongolian_YN and Mongolian_IM were more similar to each other than Mongolian_YN was to the other southern populations of China.

However, the lowest *F*_*ST*_ based on HLA-DRB1 was found between Mongolian_YN and Wa (0.011); this value is smaller than the *F*_*ST*_ between Mongolian_YN and the other populations (0.033–0.074). On the other hand, the *F*_ST_ between Mongolian_YN and Mongolian_IM was 0.054. All the paired *F*_*ST*_ values were significantly different (α = 0.002 after Bonferroni correction), except for the Mongolian_IM-Han *F*_*ST*_ (*F*_*ST*_ = 0.000, P = 0.801) and the Mongolian_YN-Wa *F*_*ST*_ (*F*_*ST*_ = 0.011, P = 0.007). These results suggest that the HLA-DRB1 frequencies are similar between Mongolian_YN and Wa, but different between Mongolian_YN and Mongolian_IM.

**Fig 2:**
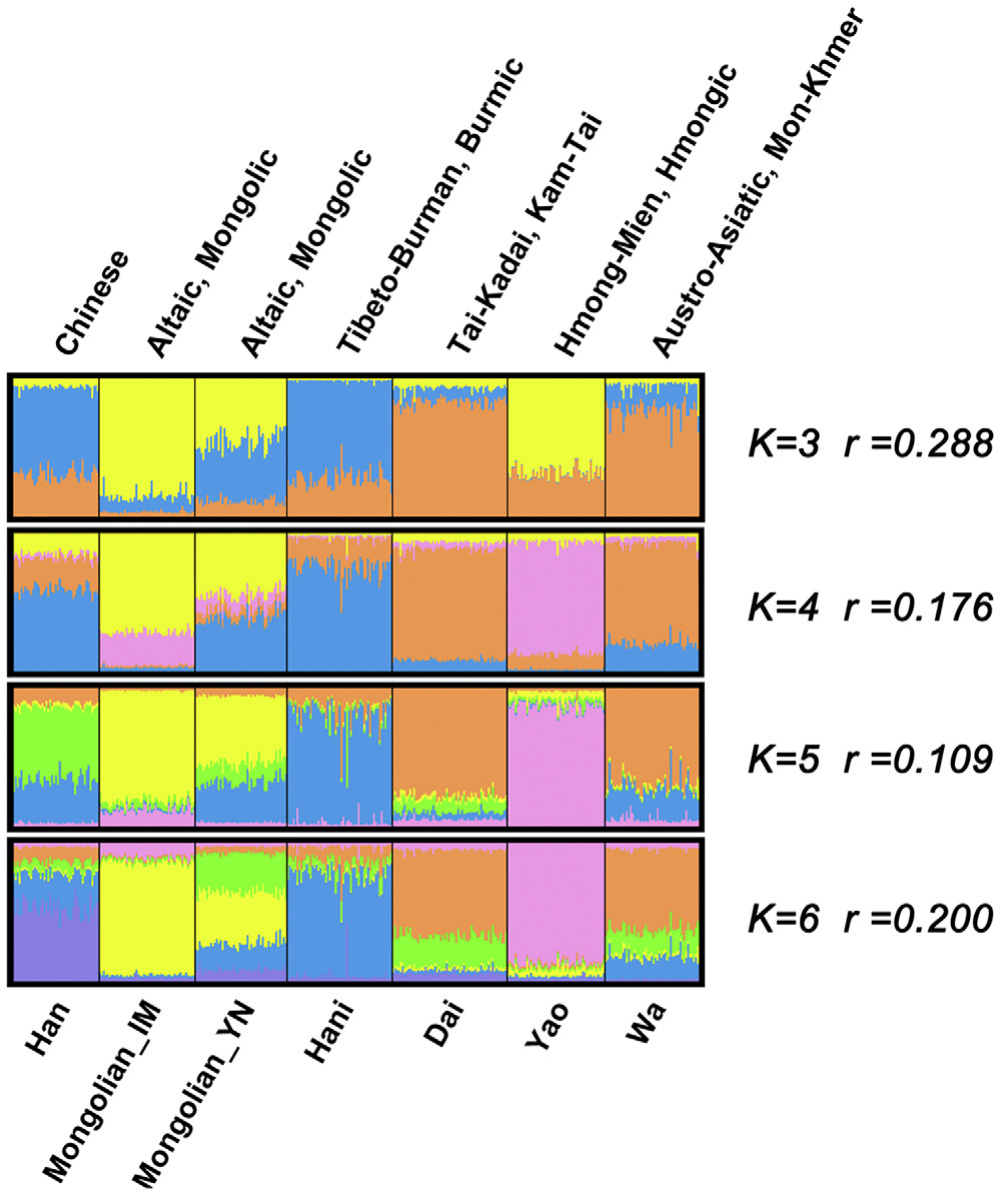
Clustering analysis from STRUCTURE. Population structure was investigated using STRUCTURE software, assuming K = 3, 4, 5, and 6. Populations were ordered according to their linguistic affiliations. Linguistic affiliations and population names are labeled above and beneath the plot. According to the guidelines in the STRUCTURE manual and ΔK values at different Ks, K = 5 is the most appropriate value. The *r* values indicate that more information is given with the LOCPRIOR model than without it.

Furthermore, a diagram of the genetic components of the seven populations based on the STR data was constructed using STRUCTURE software and is shown in Fig 2. In Fig 2, the genetic components of seven populations with K values of 3, 4, 5, and 6 are shown. We observed a plateau of the estimated posterior probability at K = 5, and ΔK also showed a maximum value at K = 5 in S1 Fig [28] Therefore, in the seven populations, five genetic components likely existed. When K = 5, approximately 50% of the genetic components (marked in yellow) of Mongolian_YN were derived from Mongolian_IM (Q = 0.489; average admixture proportion estimated with STRUCTURE software), which was consistent with the analysis results using *F*_*ST*_ values. An additional 30% of the genetic components of Mongolian_YN (marked in blue in Fig 2) was composed of another important element (average Q = 0.289). This blue component, depicted in Fig 2, was the major genetic component of the Hani people; thus, the gene flow that affected the genetic composition of Mongolian_YN might originate from Hani, or some people speaking Tibetan-Burmese and having similar a genetic background with the Hani in the south. The major genetic components of the Wa, which had similar HLA-DRB1 gene frequencies to that of Mongolian_YN, showed a rather large divergence from those of Mongolian_YN. The major genetic components of the Wa(marked in orange in Fig 2) were rarely observed in Mongolian_YN. Therefore, it looks like the population admixture maybe occurs between Mongolian_YN and Hani, rather than Mongolian_YN and Wa. An analysis of gene flow should be performed.

### Gene flow

In addition to the two Mongolian populations, two populations from the south were chosen to identify the real admixture event in the history of Mongolian_YN. The first was the Hani people because this population shares certain genetic components with Mongolian_YN. The second was the Wa, as the HLA-DRB1 allele composition of this population was similar to that of Mongolian_YN. Three scenarios were constructed and are shown in S2 Fig In the first scenario, Mongolian_YN separated from their ancestors 700 years ago and migrated to Yunnan Province but did not exchange genetic material with the indigenous people. In the second scenario, gene flow occurred between Mongolian_YN and the Hani people after Mongolian_YN immigrated to Yunnan. In the third scenario, Mongolian_YN exchanged genetic material with the Wa people after migration. These three scenarios were simulated, and their posterior probabilities were calculated using DIYABC 2.0 software[31] based on STRs. The posterior probability of each scenario was evaluated using polychotomous logistic regression on the 1% of simulated datasets closest to the observed dataset, as shown in Table 6, after 3 million simulated datasets were generated (1 million for each scenario).[18, 37] The highest posterior probability is 0.748 [95%CI: (0.645–0.850)] in scenario 2. Following the recommendations of Robert et al.,[38] we evaluated the power of the model choice procedure using the method implemented in DIYABC, and the results are listed in Table 6. For that purpose, we first simulated 500 random datasets under the selected scenario (scenario 2) and computed the proportion of cases in which this scenario did not display the highest posterior probability among all scenarios. This empirical estimate of the type I error was only13.8%. We then empirically estimated the type II error rate by simulating 500 random datasets under the other two scenarios (scenario 1 and scenario 3) and computing the proportion of cases in which scenario 2 was incorrectly selected as the most likely scenario on these simulated datasets. The type II error rate was 5%, indicating 95% statistical power. Hence, this simulation-based evaluation clearly showed that, given the size and polymorphism rate of our STR dataset, scenario 2 had sufficient power to distinguish between the alternative demographic scenarios that we investigated. Hence, gene flow should have occurred between Mongolian_YN and Hani (or some Tibetan-Burmese speakers with a similar genetic background to the Hani).

**Table 6:** Model choice and performance of the ABC analysis.

Scenario	Relative posterior probability (95% CI)	P(SC2) *	Gene-flow hypothesis
SC1	0.001 (0.000–0.035)	0.014^‡^	No gene flow among these populations
SC2	0.748 (0.645–0.850)	0.862^§^	Gene flow occurred between Mongolian_YN and Hani
SC3	0.251 (0.149–0.354)	0.036^‡^	Gene flow occurred between Mongolian_YN and Wa

*P(SC2) is the proportion of pseudo-observed simulated datasets using each competing scenario (SC1 to SC3) for which SC2 was selected because it had the highest posterior probability.

^‡^For SC1 and SC3, P(SC2) represents an empirical estimate of the model-specific type II error rate (here, 3.6%+1.4% = 5%).

^§^For SC2, 1 − P(SC2) provides an empirical estimate of the type I error rate (here, 13.8%).

**Fig 3:**
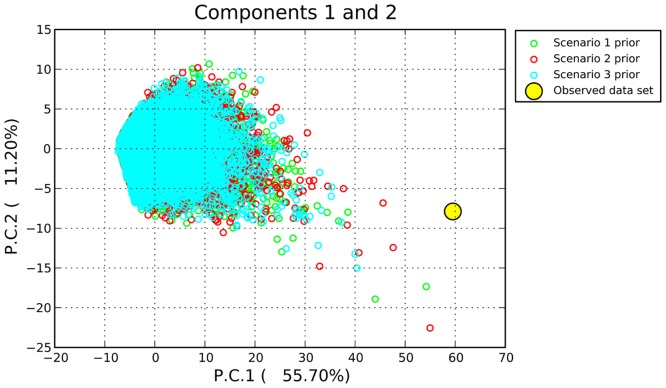
PCA of the three scenarios. The principal component analyses (PCAs) of summary statistics were performed using DIYABC 2.0 software with three different scenarios (see S2 Fig). The summary statistics of the different scenarios were calculated based on the computer simulation of HLA-DRB1 sequences under widely predefined demographic parameters. The observed data represent summary statistics of real HLA-DRB1 sequence data.

Although gene flow has had an impact on the frequencies of HLA-DRB1, we still wanted to know whether gene flow itself was able to shape the frequency distribution of HLA-DRB1 alleles in the current Mongolian_YN population when selective pressure was absent. The simulation was therefore repeated 500,000 times in DIYABC using the HLA-DRB1sequence data, followed by PCA to compare the differences in summary statistics between the simulated value and the observed value. The results of this comparison are shown in Fig 3. All simulated data were considerably different from the observed data, suggesting that none of the three scenarios (including the true scenario, scenario 2) were capable of explaining the current HLA-DRB1 composition in Mongolian_YN. Therefore, we believe that inter-population gene flow alone was not responsible for shaping the HLA-DRB1 gene pattern of Mongolian_YN and that these changes were driven by selective pressure.

### Allele frequencies of HLA-DRB1

**Fig 4:**
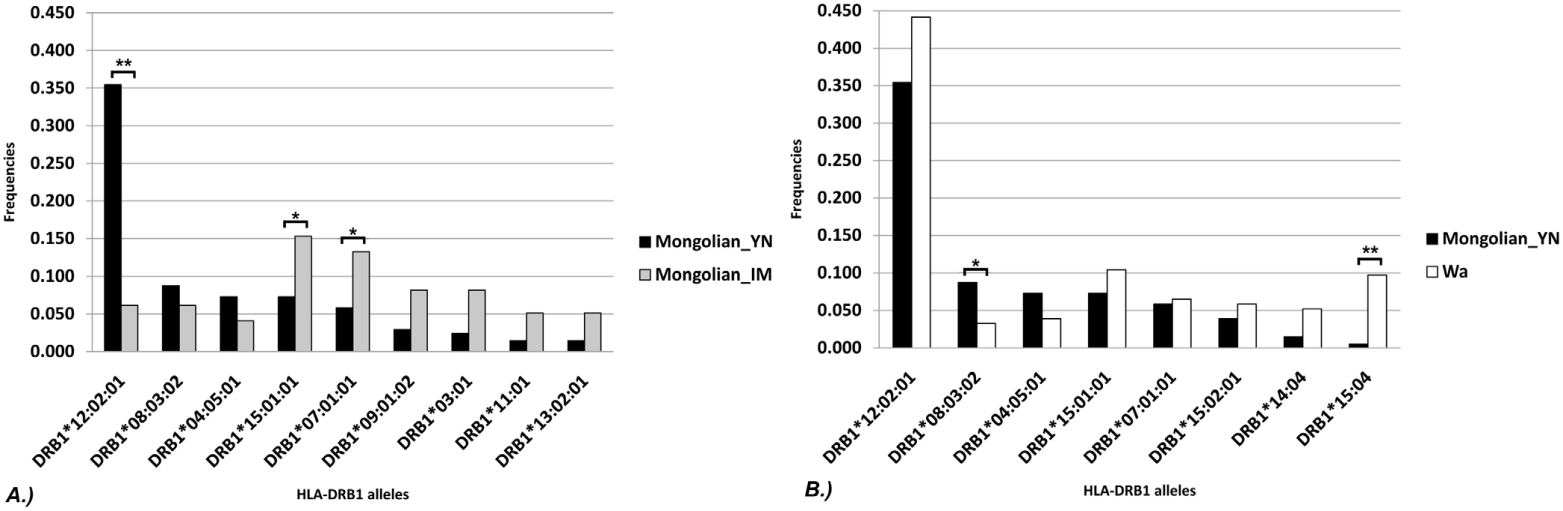
Comparisons of allele frequencies. Allele frequencies higher than 5% in any one of the populations were compared between A.) Mongolian_YN and Mongolian_IM or B.) Mongolian_YN and Wa. ** indicates P< 0.0001 using a 2×2 χ^2^ test. * indicates P< 0.05.

HLA-DRB1 in Mongolian_YN was subjected to the influence of selection following the migration into Yunnan, and the details of this change merit study. Thus, the composition ratios of the HLA-DRB1 alleles of the three populations were compared with each other using the R × C chi-squared test. As shown in Table 7, the R × C chi-squared test revealed significant differences (P<0.0001) between the composition ratios of the alleles of Mongolian_YN and Mongolian_IM. As seen in Fig 4, after Mongolian_YN moved from Mongolia to Yunnan, the DRB1*12:02:01 allele frequency increased by approximately six-fold, from 6.1% to 35.4% (2 × 2 χ^2^ = 29.7, P <0.0001). The DRB1*12:02:01 allele was common in the southern populations, which contained large proportions of the Wa, Hani and Dai people, with a gene frequency in the range of 16.9–44.2%. However, in Mongolian_YN (people immigrated to Yunnan from Mongolia), the gene frequencies of DRB1*07:01:01 and DRB1*15:01:01 decreased by approximately half. The frequency of DRB1*07:01:01 decreased from 13.3% to 5.8% (2 × 2 χ^2^ = 4.87, P = 0.027), whereas that of DRB1*15:01:01 decreased from 15.3% to 7.3% (2 × 2 χ^2^ = 4.81, P = 0.028).

**Table 7:** The difference in the frequency of HLA-DRB1 alleles between the Mongolian population in Yunnan and other populations.

HLA-DRB1 Alleles	Number of HLA-DRB1 Alleles			Number of HLA-DRB1 Alleles		
	Mongolian_YN(%)	Mongolian_IM(%)	χ^2^ *&P* *	Mongolian_YN(%)	Wa(%)	*χ* ^*2*^ *&P* *
DRB1*04:05:01	15(7.3%)	4(4.1%)		15(7.3%)	6(3.9%)	
DRB1*07:01:01	12(5.8%)	13(13.3%)		12(5.8%)	10(6.5%)	
DRB1*08:03:02	18(8.7%)	6(6.1%)		18(8.7%)	5(3.2%)	
DRB1*12:02:01	73(35.4%)	6(6.1%)		73(35.4%)	68(44.2%)	
DRB1*15:01:01	15(7.3%)	15(15.3%)		15(7.3%)	16(10.4%)	
^#^LowFreqAlleles	73(35.4%)	54(55.1%)	χ^2^ = 38.6	73(35.4%)	49(31.8%)	χ^2^ = 8.99
Total	206(100.0%)	98(100.0%)	P<0.0001	206(100.0%)	154(100.0%)	P = 0.109

*R×C χ^2^ test between the two listed populations.

^#^LowFreqAlleles: Merged alleles with frequencies lower than 5% in Mongolian_IM.

The result of the comparison between Mongolian_YN and the Wa people was consistent with the results of the *F*_*ST*_ analysis. The *F*_*ST*_ value between these two populations is not significant (*F*_*ST*_ = 0.011, P = 0.007). As seen in S5 Table, there was no significant difference (P = 0.109) between the allele composition ratio of HLA-DRB1 alleles in Mongolian_YN and that in Wa. However, it is worth noting that the DRB1*15:04 allele accounted for 9.7% of the alleles in the Wa population, whereas it only accounted for 0.5% in Mongolian_YN (2 × 2 χ^2^ = 17.8, P<0.0001).

## Discussion

Previous studies have reported on HLA-DRB1 genotype in certain populations, such as Wa, Dai and Yao, using PCR-SSOP methods[39]. Similar allele frequencies were obtained using our sequencing methods. Based on our data, we found that the gene frequencies of some alleles changed noticeably in the two Mongolian populations. The frequency of the DRB1*12:02:01 allele, which was low in Mongolian_IM, increased by six-fold in Mongolian_YN, from 6.1% to 35.4%, whereas the frequencies of DRB1*07:01:01 and DRB1*15:01:01, which were relatively high in Mongolian_IM, decreased by approximately half. The HLA-DRB1 alleles DRB1*07:01:01 and DRB1*15:01:01 were predominant in the two northern populations in this study, and their frequencies were also high in the other northern populations of China.[39, 40] In contrast, the DRB1*12:02:01 allele was widespread in the southern populations investigated in this study (except for the Yao people) and in other reports, such as the Lahu, Lisu, Naxi, and Nu.[39] It appears that the “northern pattern” of the HLA-DRB1 frequency spectrum suddenly changed into “southern pattern” in less than 800 years, and this finding is also confirmed by the *F*_*ST*_ analysis and R × C chi-squared test. The *F*_*ST*_ based on HLA-DRB1 between the Mongolian_YN and Wa populations was not statistically significant (*F*_*ST*_ = 0.011, P = 0.007), whereas the *F*_*ST*_ based on HLA-DRB1 between Mongolian_YN and Mongolian_IM was significant (*F*_*ST*_ = 0.054, P < 0.0001). The R × C chi-squared test also showed a significant difference between the HLA frequencies in Mongolian_YN and Mongolian_IM (χ2 = 38.6, P <0.0001), whereas the difference is not significant between Mongolian_YN and Wa (χ2 = 8.99, P = 0.109).

We are very interested in understanding which force was responsible for changing the HLA-DRB1 frequencies so dramatically. Both demographic events and natural selection can alter the gene frequencies in a population. First, the demographic events that the Mongolian_YN experienced were investigated. Genetic drift and gene flow are two important demographic forces that can remodel gene frequencies. Because population expansion and reduction were excluded by a bottleneck analysis based on neutral STRs, genetic drift appears not to be the force that we are looking for. Thus, 10 neutral STRs were chosen to analyze the genetic backgrounds of the seven populations to identify potential gene flow. Using the STRUCTURE program, we found that Mongolian_YN and Mongolian_IM shared 50% genetic similarity. However, approximately 30% of the genetic component of Mongolian_YN might originate from southern Tibeto-Burman speaking people such as the Hani. Thus, gene flow from the local population of southern China has clearly had an impact on the genetic background of Mongolian_YN. However, certain aspects of this gene frequency are difficult to account for by gene flow alone. One factor is the high frequency of DRB1*12:02:01 in Mongolian_YN. DRB1*12:02:01 is a common variant in the southern populations of China. Yet the frequencies of DRB1*12:02:01 in the populations with the potential to exchange genes with Mongolian_YN were lower than that in Mongolian_YN. The frequency of DRB1*12:02:01 is 20.9% in the Hani people, and in other Tibeto-Burman speakers, the DRB1*12:02:01 frequencies are approximately 30%.[39] Because the frequencies of DRB1*12:02:01 were not very high, it is difficult to believe that gene flow from other local populations could have increased the frequency of DRB1*12:02:01 in Mongolian_YN to 35.4%. Therefore, to verify that the current gene composition of HLA-DRB1 in Mongolian_YN was not caused by inter-population gene flow alone, we used the DIYABC program to simulate the formation of the HLA-DRB1 gene composition in Mongolian_YN in the absence of selective pressure, with genetic composition dependent only on gene flow. Although less stringent demographic parameters were chosen in the simulation, the simulated values diverged from the observed values even in the true scenario, which was demonstrated by DIYABC analysis based on STRs. Therefore, it is very unlikely that the current genetic composition of HLA-DRB1 formed solely through inter-population gene flow.

Thus, we can conclude that natural selection took place in Mongolian_YN after the Mongols came to southern China roughly 700 years ago. Most researchers believe that balancing selection maintains a high degree of diversity in MHC, whereas pathogenic drive is one of the major factors responsible for maintaining this selective pressure.[3, 4] Given the different pathogen spectra that exists in southern China [7, 41], another interesting question is whether the impact o selection on Mongolian_YN and Mongolian_IM are same. To examine this, the parameter based on the site frequency spectrum, Tajima's *D*, was calculated. In the seven populations, all values for Tajima's *D* based on the sequences of HLA-DRB1 exon 2were larger than 0. Excluding the impact of the bottleneck effect caused by demographic events, we believe that exon 2 of HLA-DRB1 in all seven populations was affected by balancing selection. There were no significant differences in Tajima's *D* between the northern and southern populations, and the Tajima's *D* for Mongolian_YN (2.96) and Tajima's *D* for Mongolian_IM (2.93) were also similar. Another analysis was performed based on dN/dS using the PAML program. The results indicated that approximately 12% of sites were subjected to positive selection in both Mongolian populations, and there was no significant difference between the two populations. From the above results, it appears that although Mongolian_YN was challenged with a new pathogen spectrum after immigration, the types of selection exerted on HLA-DRB1 and the number of sites that enabled the selective force in the population did not change.

However, these findings do not necessarily mean that HLA-DRB1 is under similar selective pressures in both the northern and the southern Mongolians and that there is no significant difference of pathogen spectrum between northern and southern China. If Mongolian_YN underwent a similar balancing selection as Mongolian_IM, it is hard to explain why the frequency spectrum of Mongolian_YN did not show an excess of intermediate-frequency alleles, as is the case in Mongolian_IM. Furthermore, the dramatic increase in the DRB1*12:02:01 frequency in Mongolian_YN is also hard to explain with respect to similar balancing selection in the south and north of China. Therefore, we investigated the sites under positive selection using EBE analysis in PAML software. We found that the sites under positive selection were different in the two Mongolian populations. The 57^th^ amino acid was positively selected in the Mongolian_YN but not in the Mongolian_IM. On the other hand, the 74^th^ amino acid was positively selected in the Mongolian_IM but not in the Mongolian_YN. Furthermore, the 57^th^ amino acid of DRB1*12:02:01 is valine, whereas it is aspartic acid, serineor alanine in the proteins which other HLA-DRB1 alleles code. The positive selection maybe impact on these two sites and push DRB1*12:02:01 to a higher frequency.

Based on these data, we propose the following model. HLA-DRB1 is known to be under balancing selection. The balancing selection is a heterogeneity action in a period time. In a way, this selection encompasses a number of types of selection.[42] MHC genes have been predicted to be under very long-term balancing selection. This balancing selection is actually composed of many purifying/ positive selections over a short time. This balancing selection leads to the accumulation of DNA mutations in the HLA-DRB1 gene and keeps the HLA-DRB1 gene polymorphic. Therefore, when using statistical parameters based on DNA mutations (such as Tajima’s *D* or dN/dS) to test natural selection, we obtain information on selection over a long period of time. Southern Mongolians only separated from northern Mongolian less than 800 years ago. DNA mutations have not had enough time to occur and become fixed in these populations. Therefore, when we used analyses based on DNA mutations, we found that northern and southern Mongolians appear to be under similar selective pressure and that the proportions of sites under selection are similar. In fact, in response to changes in the pathogens spectrum over such a short time, natural selection has already been acting on the different sites of HLA-DRB1 exon 2. This has led to increased frequency for some allele that already have existed in the population, such as DRB1*12:02:01. Therefore, we believed that positive selection impacted on some sites of HLA-DRB1 and altered the gene frequency in Mongolian_YN. The differences in HLA-DRB1 allele frequencies between Mongolian populations are adaptive.

Comparing the composition of the pathogen spectrum of southern and northern China, the prevalence of malaria differed significantly. Malaria was very rare in the north, with high rates in the south, particularly to the south of 25°Nlatitude. The frequency of malarial infection was approximately 0.1% in the past 50 years, and the high incidence of the disease was caused by *Plasmodium falciparum*.[41] Mutations in glucose 6-phosphate dehydrogenase (G6PD) driven by selective pressure from malaria are known to be very common in southern China, especially to the south of 25°N latitude.[43] Therefore, we speculate that the selective pressure exerted by malaria may also have affected the gene frequencies of HLA-DRB1 in Mongolian_YN.

Individuals carrying the DRB1*12:01 allele have been reported to produce a high level of immune antibodies against *Plasmodium falciparum* asexual-stage apical membrane antigen 1 (AMA1).[44] However, individuals carrying DRB1*15 only produce a low level of antibodies against the malaria vaccine SPF66. These data support the observation that DRB1*12:02: 01 increased and DRB1*15:01:01 decreased in Mongolian_YN. Nevertheless, establishing the relationship between these HLA-DRB1 alleles and malaria warrants further validation through additional functional experiments.

## Supporting Information

S1 Fig Best fit K estimation. A.) Mean likelihood L(K) value and variance per K value were calculated from five runs of STRUCTURE. When K = 5, the likelihood value was highest. B.) The ΔK plot, which was suggested by Evanno et al. (2005), was used to detecting the number of K groups that best fit the data. When K = 5, ΔK was highest.
(TIF)

S2 Fig Alternative scenarios for ABC analysis. To address the possibility that the allele distributions of HLA-DRB1 in Mongolian_YN may be only due to gene flow, three scenarios were constructed. Scenario 1: after the Mongolian people came into Yunnan, gene flow with other populations did not occur. Scenario 2: gene flow occurred between Mongolian_YN and Hani after Mongolian southern migration. Scenario 3: gene flow occurred between Mongolian_YN and Wa after Mongolian southern migration. Details of the parameters used in each scenario are provided in the Materials and Methods section.
(TIF)

S1 Table The allele frequencies, expected *he*terozygosities (*He*), observed *he*terozygosities (*Ho*), fixation index (*F* = (*He*—*Ho*)/*He*) and Hardy-Weinberg equilibrium (HWE) tests of the seven ethnic groups for HLA-DRB1. (DOC)

S2 Table The allele frequencies, expected *he*terozygosities (*He*), observed *he*terozygosities (*Ho*), fixation index (*F* = (*He*—*Ho*)/*He*) and Hardy-Weinberg equilibrium (HWE) tests of the seven ethnic groups for the 10 microsatellites. (DOC)

S3 Table Likelihood values and parameter estimates for HLA-DRB1 exon 2 in the five populations. (DOC)

S4 Table Population pairwise *F*_ST_ values based on STR analysis. (DOC)

S5 Table Population pairwise *F*_ST_ values based on alleles frequencies of HLA-DRB1. (DOC)
