# Supplementary figures and images for: The Adaptive Change of HLA-DRB1 Allele Frequencies Caused by Natural Selection in a Mongolian Population That Migrated to the South of China
Source: PLoS One. 2015 Jul 31;10(7):e0134334. doi: 10.1371/journal.pone.0134334 (PMC4521750; doi:10.1371/journal.pone.0134334)

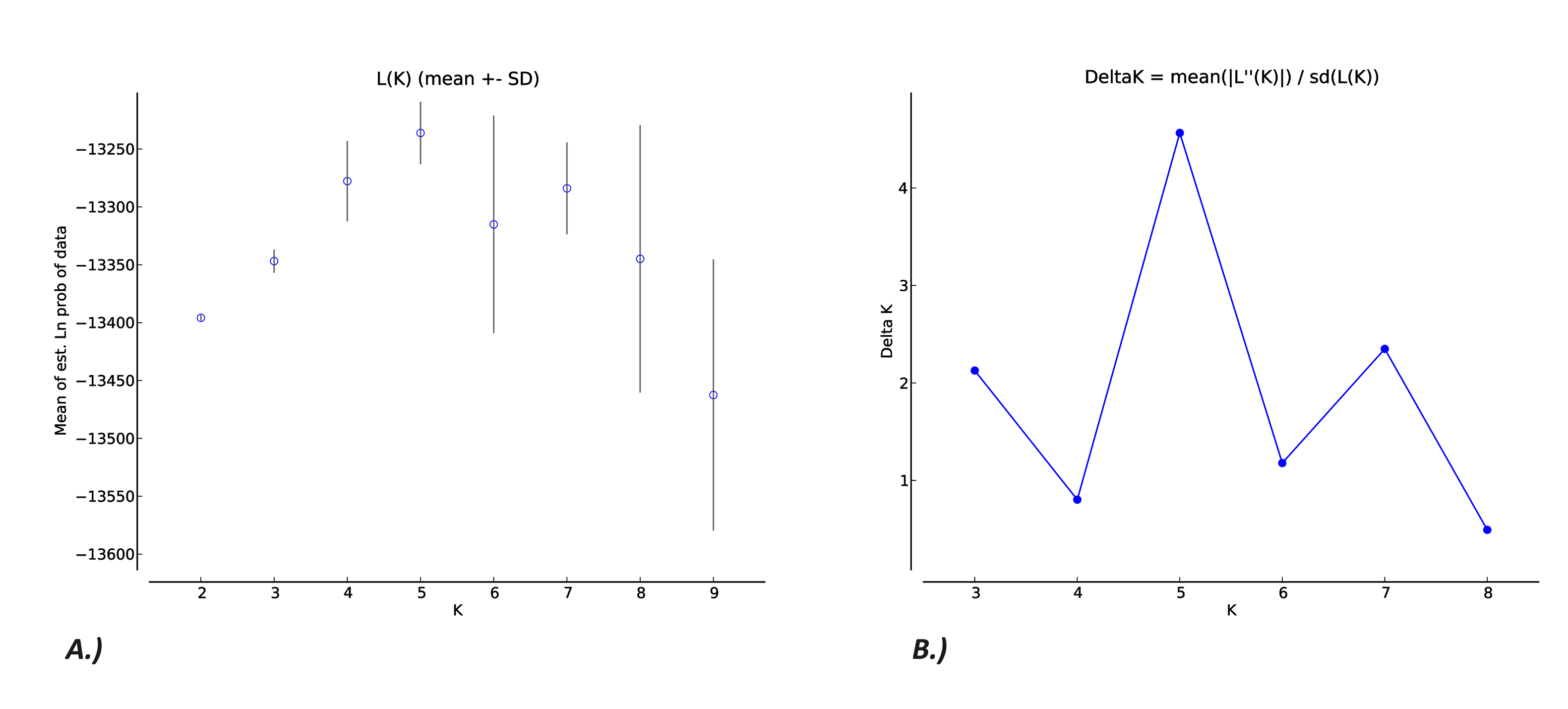

Supplement: S1 Fig — A.) Mean likelihood L(K) value and variance per K value were calculated from five runs of STRUCTURE. When K = 5, the likelihood value was highest. B.) The ΔK plot, which was suggested by Evanno et al. (2005), was used to detecting the number of K groups that best fit the data. When K = 5, ΔK was highest. (TIF) [file pone.0134334.s001.tif]

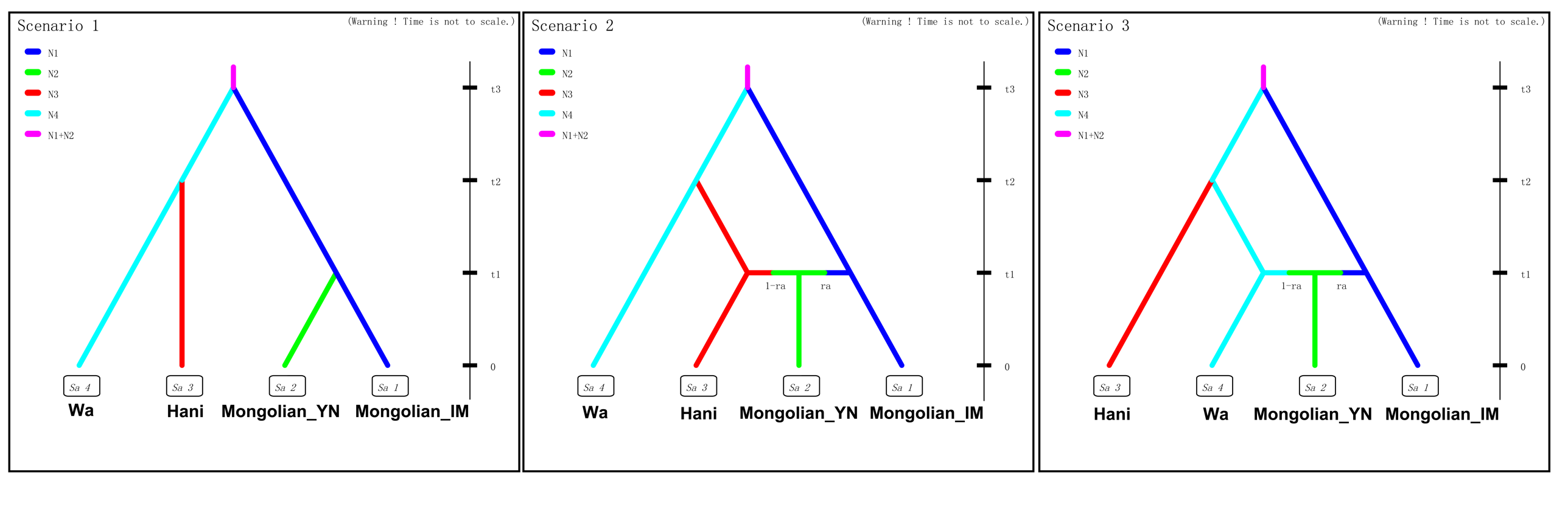

Supplement: S2 Fig — To address the possibility that the allele distributions of HLA-DRB1 in Mongolian_YN may be only due to gene flow, three scenarios were constructed. Scenario 1: after the Mongolian people came into Yunnan, gene flow with other populations did not occur. Scenario 2: gene flow occurred between Mongolian_YN and Hani after Mongolian southern migration. Scenario 3: gene flow occurred between Mongolian_YN and Wa after Mongolian southern migration. Details of the parameters used in each scenario are provided in the Materials and Methods section. (TIF) [file pone.0134334.s002.tif]
