# Supplementary material for: The Adaptive Change of HLA-DRB1 Allele Frequencies Caused by Natural Selection in a Mongolian Population That Migrated to the South of China
Source: PLoS One. 2015 Jul 31;10(7):e0134334. doi: 10.1371/journal.pone.0134334 (PMC4521750; doi:10.1371/journal.pone.0134334)
Supplement: S1 Table — (DOC) [file pone.0134334.s003.doc]

**Table S1: The allele frequencies, expected *he*terozygosities (*He*), observed *he*terozygosities (*Ho*), fixation index (*F*= (*He* - *Ho*)/*He***) and Hardy-Weinberg equilibrium (HWE) tests of the seven ethnic groups for HLA-DRB1

| **Allele**  **(*He*, *Ho*, *F*, HWE)** | **Han**  **(n=98)** | **Mongolian_IM**  **(n=98)** | **Mongolian_YN**  **(n=206)** | **Hani**  **(n=134)** | **Dai**  **(n=130)** | **Yao**  **(n=120)** | **Wa**  **(n=154)** |
| --- | --- | --- | --- | --- | --- | --- | --- |
| **DRB1*03:01****^#^** | 0.051 | 0.082 | 0.024 | 0.075 | 0.015 | 0.058 | 0.006 |
| **DRB1*04:06^#^** | 0.020 | 0.010 | 0.005 | 0.000 | 0.008 | 0.000 | 0.006 |
| **DRB1*08:01^#^** | 0.000 | 0.010 | 0.000 | 0.000 | 0.000 | 0.000 | 0.000 |
| **DRB1*08:28** | 0.000 | 0.000 | 0.005 | 0.000 | 0.000 | 0.000 | 0.000 |
| **DRB1*11:01^#^** | 0.031 | 0.051 | 0.015 | 0.015 | 0.015 | 0.017 | 0.006 |
| **DRB1*11:04^#^** | 0.000 | 0.010 | 0.000 | 0.000 | 0.000 | 0.000 | 0.000 |
| **DRB1*14:04** | 0.000 | 0.000 | 0.015 | 0.090 | 0.038 | 0.000 | 0.052 |
| **DRB1*14:10** | 0.000 | 0.000 | 0.000 | 0.000 | 0.023 | 0.000 | 0.000 |
| **DRB1*15:04** | 0.000 | 0.000 | 0.005 | 0.022 | 0.038 | 0.000 | 0.097 |
| **DRB1*01:01:01** | 0.000 | 0.031 | 0.010 | 0.000 | 0.008 | 0.000 | 0.000 |
| **DRB1*01:02:01** | 0.010 | 0.000 | 0.000 | 0.000 | 0.000 | 0.000 | 0.000 |
| **DRB1*04:01:01** | 0.000 | 0.031 | 0.010 | 0.000 | 0.023 | 0.000 | 0.000 |
| **DRB1*04:03:01** | 0.020 | 0.000 | 0.000 | 0.007 | 0.000 | 0.008 | 0.006 |
| **DRB1*04:04:01** | 0.000 | 0.000 | 0.010 | 0.000 | 0.000 | 0.000 | 0.000 |
| **DRB1*04:05:01** | 0.061 | 0.041 | 0.073 | 0.000 | 0.031 | 0.092 | 0.039 |
| **DRB1*04:07:01** | 0.000 | 0.010 | 0.000 | 0.000 | 0.000 | 0.000 | 0.000 |
| **DRB1*04:10:01** | 0.010 | 0.000 | 0.005 | 0.000 | 0.000 | 0.000 | 0.000 |
| **DRB1*07:01:01** | 0.173 | 0.133 | 0.058 | 0.000 | 0.008 | 0.017 | 0.065 |
| **DRB1*08:02:01** | 0.020 | 0.010 | 0.010 | 0.000 | 0.000 | 0.000 | 0.000 |
| **DRB1*08:03:02** | 0.041 | 0.061 | 0.087 | 0.052 | 0.023 | 0.075 | 0.032 |
| **DRB1*09:01:02** | 0.102 | 0.082 | 0.029 | 0.022 | 0.069 | 0.025 | 0.013 |
| **DRB1*10:01:01** | 0.000 | 0.031 | 0.005 | 0.000 | 0.008 | 0.008 | 0.000 |
| **DRB1*11:06:01** | 0.000 | 0.000 | 0.005 | 0.000 | 0.038 | 0.000 | 0.013 |
| **DRB1*12:01:01** | 0.031 | 0.020 | 0.010 | 0.000 | 0.000 | 0.008 | 0.000 |
| **DRB1*12:02:01** | 0.102 | 0.061 | 0.354 | 0.209 | 0.169 | 0.050 | 0.442 |
| **DRB1*13:01:01** | 0.010 | 0.020 | 0.019 | 0.000 | 0.023 | 0.000 | 0.045 |
| **DRB1*13:02:01** | 0.051 | 0.051 | 0.015 | 0.000 | 0.031 | 0.000 | 0.006 |
| **DRB1*13:02:06** | 0.000 | 0.010 | 0.000 | 0.000 | 0.000 | 0.000 | 0.000 |
| **DRB1*13:12:01** | 0.000 | 0.000 | 0.024 | 0.000 | 0.008 | 0.042 | 0.000 |
| **DRB1*14:03:01** | 0.020 | 0.010 | 0.019 | 0.000 | 0.000 | 0.000 | 0.000 |
| **DRB1*14:05:01** | 0.010 | 0.000 | 0.024 | 0.000 | 0.008 | 0.008 | 0.000 |
| **DRB1*14:12:01** | 0.000 | 0.010 | 0.000 | 0.000 | 0.000 | 0.000 | 0.000 |
| **DRB1*14:54:01** | 0.020 | 0.031 | 0.039 | 0.306 | 0.069 | 0.208 | 0.006 |
| **DRB1*15:01:01** | 0.143 | 0.153 | 0.073 | 0.097 | 0.085 | 0.175 | 0.104 |
| **DRB1*15:01:04** | 0.000 | 0.000 | 0.000 | 0.000 | 0.008 | 0.000 | 0.000 |
| **DRB1*15:02:01** | 0.061 | 0.020 | 0.039 | 0.104 | 0.154 | 0.133 | 0.058 |
| **DRB1*15:02:02** | 0.000 | 0.000 | 0.000 | 0.000 | 0.008 | 0.000 | 0.000 |
| **DRB1*16:01:01** | 0.010 | 0.000 | 0.000 | 0.000 | 0.000 | 0.000 | 0.000 |
| **DRB1*16:02:01** | 0.000 | 0.020 | 0.015 | 0.000 | 0.092 | 0.075 | 0.000 |
| ***H*o** | 0.878 | 0.939 | 0.883 | 0.881 | 0.969 | 0.917 | 0.831 |
| ***H*e** | 0.919 | 0.935 | 0.849 | 0.831 | 0.920 | 0.887 | 0.774 |
| ***F*** | 0.046 | -0.005 | -0.041 | -0.060 | -0.054 | -0.034 | -0.074 |
| **p of HWE** | 0.119 | 0.778 | 0.507 | 0.097 | 0.062 | 0.990 | 0.715 |

n: number of chromosomes.

#: These alleles included ambiguous alleles. Ambiguous alleles shared the same sequence of HLA-DRB1 exon 2 were seen as a variant to performed selection analyses, because they were under a same selection pressure. DRB*03:01 included ambiguous alleles DRB1*03:01:01:01, DRB1*03:01:01:02 and DRB1*03:01:08; DRB*04:06 included ambiguous alleles DRB1*04:06:01 and DRB1*04:06:02; DRB1*08:01 included ambiguous alleles DRB1*08:01:01 and DRB1*08:01:03; DRB1*11:01 included ambiguous alleles DRB1*11:01:01 and DRB1*11:01:06; DRB1*11:04 included ambiguous alleles DRB1*11:04:01, DRB1*11:04:02, DRB1*11:04:06 and DRB1*11:04:09.
