# Supplementary material for: The Adaptive Change of HLA-DRB1 Allele Frequencies Caused by Natural Selection in a Mongolian Population That Migrated to the South of China
Source: PLoS One. 2015 Jul 31;10(7):e0134334. doi: 10.1371/journal.pone.0134334 (PMC4521750; doi:10.1371/journal.pone.0134334)
Supplement: S2 Table — (DOC) [file pone.0134334.s004.doc]

**Table S2. The allele frequencies, expected *he*terozygosities (*He*), observed *he*terozygosities (*Ho*), fixation index (*F*= (*He* - *Ho*)/*He***) and Hardy-Weinberg equilibrium (HWE) tests of the seven ethnic groups for the 10 microsatellites

| **Locus** | **Allele(*He*, *Ho*, *F*, HWE)** | **Han**  **(n=90)** | **Mongolian_IM**  **(n=100)** | **Mongolian_YN***  **(n=96)** | **Hani**  **(n=110)** | **Dai**  **(n=120)** | **Yao**  **(n=102)** | **Wa**  **(n=98)** |
| --- | --- | --- | --- | --- | --- | --- | --- | --- |
| **D3S1263** | **192** | 0.011 | 0.020 | 0.010 | 0.000 | 0.025 | 0.020 | 0.031 |
|  | **194** | 0.056 | 0.050 | 0.021 | 0.036 | 0.017 | 0.137 | 0.031 |
|  | **196** | 0.111 | 0.210 | 0.115 | 0.064 | 0.100 | 0.118 | 0.143 |
|  | **198** | 0.267 | 0.170 | 0.198 | 0.236 | 0.192 | 0.176 | 0.214 |
|  | **200** | 0.067 | 0.060 | 0.177 | 0.155 | 0.058 | 0.059 | 0.051 |
|  | **202** | 0.067 | 0.030 | 0.042 | 0.118 | 0.083 | 0.098 | 0.071 |
|  | **204** | 0.144 | 0.120 | 0.104 | 0.055 | 0.108 | 0.167 | 0.102 |
|  | **206** | 0.100 | 0.160 | 0.156 | 0.073 | 0.158 | 0.108 | 0.071 |
|  | **208** | 0.089 | 0.040 | 0.042 | 0.109 | 0.042 | 0.029 | 0.112 |
|  | **210** | 0.044 | 0.030 | 0.000 | 0.064 | 0.050 | 0.000 | 0.122 |
|  | **212** | 0.022 | 0.060 | 0.031 | 0.073 | 0.108 | 0.029 | 0.031 |
|  | **214** | 0.011 | 0.030 | 0.073 | 0.018 | 0.017 | 0.049 | 0.010 |
|  | **216** | 0.011 | 0.020 | 0.031 | 0.000 | 0.042 | 0.010 | 0.010 |
|  | ***Ho*** | 0.689 | 0.880 | 0.875 | 0.764 | 0.900 | 0.961 | 0.857 |
|  | ***He*** | 0.873 | 0.881 | 0.879 | 0.879 | 0.895 | 0.888 | 0.889 |
|  | ***F*** | 0.212 | 0.001 | 0.005 | 0.132 | -0.006 | -0.083 | 0.036 |
|  | **p of HWE** | 0.000 | 0.577 | 0.615 | 0.075 | 0.201 | 0.126 | 0.100 |
|  |  |  |  |  |  |  |  |  |
| **D3S1266** | **290** | 0.011 | 0.000 | 0.000 | 0.000 | 0.000 | 0.000 | 0.000 |
|  | **294** | 0.422 | 0.390 | 0.333 | 0.278 | 0.233 | 0.196 | 0.235 |
|  | **296** | 0.078 | 0.160 | 0.083 | 0.139 | 0.083 | 0.186 | 0.153 |
|  | **298** | 0.044 | 0.060 | 0.021 | 0.056 | 0.067 | 0.088 | 0.102 |
|  | **300** | 0.000 | 0.020 | 0.000 | 0.019 | 0.000 | 0.000 | 0.000 |
|  | **302** | 0.389 | 0.310 | 0.479 | 0.481 | 0.558 | 0.422 | 0.469 |
|  | **304** | 0.033 | 0.060 | 0.083 | 0.028 | 0.042 | 0.108 | 0.041 |
|  | **306** | 0.011 | 0.000 | 0.000 | 0.000 | 0.000 | 0.000 | 0.000 |
|  | **308** | 0.011 | 0.000 | 0.000 | 0.000 | 0.017 | 0.000 | 0.000 |
|  | ***Ho*** | 0.689 | 0.780 | 0.646 | 0.667 | 0.617 | 0.686 | 0.735 |
|  | ***He*** | 0.668 | 0.726 | 0.652 | 0.674 | 0.626 | 0.737 | 0.696 |
|  | ***F*** | -0.031 | -0.075 | 0.009 | 0.011 | 0.014 | 0.069 | -0.056 |
|  | **p of HWE** | 0.801 | 0.665 | 0.947 | 0.328 | 0.066 | 0.430 | 0.699 |
|  |  |  |  |  |  |  |  |  |
| **D3S1278** | **229** | 0.011 | 0.010 | 0.010 | 0.000 | 0.017 | 0.040 | 0.000 |
|  | **231** | 0.444 | 0.357 | 0.375 | 0.400 | 0.367 | 0.210 | 0.469 |
|  | **233** | 0.167 | 0.163 | 0.135 | 0.182 | 0.275 | 0.210 | 0.112 |
|  | **235** | 0.022 | 0.031 | 0.042 | 0.055 | 0.008 | 0.000 | 0.010 |
|  | **237** | 0.033 | 0.051 | 0.021 | 0.036 | 0.067 | 0.120 | 0.112 |
|  | **239** | 0.000 | 0.041 | 0.000 | 0.018 | 0.008 | 0.000 | 0.010 |
|  | **241** | 0.244 | 0.173 | 0.302 | 0.173 | 0.158 | 0.320 | 0.163 |
|  | **243** | 0.022 | 0.041 | 0.031 | 0.055 | 0.042 | 0.010 | 0.051 |
|  | **245** | 0.000 | 0.020 | 0.000 | 0.000 | 0.017 | 0.020 | 0.000 |
|  | **247** | 0.011 | 0.041 | 0.000 | 0.009 | 0.025 | 0.000 | 0.010 |
|  | **249** | 0.033 | 0.020 | 0.010 | 0.045 | 0.008 | 0.070 | 0.031 |
|  | **251** | 0.011 | 0.031 | 0.052 | 0.009 | 0.008 | 0.000 | 0.010 |
|  | **253** | 0.000 | 0.020 | 0.010 | 0.000 | 0.000 | 0.000 | 0.020 |
|  | **255** | 0.000 | 0.000 | 0.010 | 0.000 | 0.000 | 0.000 | 0.000 |
|  | **257** | 0.000 | 0.000 | 0.000 | 0.018 | 0.000 | 0.000 | 0.000 |
|  | ***Ho*** | 0.667 | 0.776 | 0.833 | 0.691 | 0.783 | 0.680 | 0.694 |
|  | ***He*** | 0.719 | 0.813 | 0.751 | 0.774 | 0.764 | 0.796 | 0.731 |
|  | ***F*** | 0.074 | 0.047 | -0.110 | 0.108 | -0.026 | 0.147 | 0.051 |
|  | **p of HWE** | 0.138 | 0.183 | 0.053 | 0.086 | 0.589 | 0.406 | 0.506 |
|  |  |  |  |  |  |  |  |  |
| **D3S1279** | **255** | 0.000 | 0.000 | 0.010 | 0.000 | 0.000 | 0.000 | 0.010 |
|  | **257** | 0.000 | 0.000 | 0.000 | 0.000 | 0.000 | 0.000 | 0.010 |
|  | **261** | 0.000 | 0.000 | 0.000 | 0.009 | 0.000 | 0.000 | 0.000 |
|  | **263** | 0.000 | 0.000 | 0.000 | 0.009 | 0.000 | 0.010 | 0.000 |
|  | **265** | 0.000 | 0.000 | 0.000 | 0.009 | 0.000 | 0.000 | 0.010 |
|  | **267** | 0.057 | 0.010 | 0.021 | 0.091 | 0.000 | 0.000 | 0.042 |
|  | **269** | 0.227 | 0.200 | 0.240 | 0.155 | 0.233 | 0.176 | 0.219 |
|  | **271** | 0.352 | 0.400 | 0.438 | 0.373 | 0.500 | 0.520 | 0.385 |
|  | **273** | 0.045 | 0.050 | 0.010 | 0.100 | 0.125 | 0.127 | 0.052 |
|  | **275** | 0.000 | 0.070 | 0.010 | 0.000 | 0.008 | 0.088 | 0.042 |
|  | **277** | 0.045 | 0.150 | 0.083 | 0.009 | 0.008 | 0.029 | 0.010 |
|  | **279** | 0.136 | 0.070 | 0.125 | 0.073 | 0.083 | 0.039 | 0.156 |
|  | **281** | 0.091 | 0.040 | 0.042 | 0.082 | 0.017 | 0.010 | 0.031 |
|  | **283** | 0.023 | 0.000 | 0.021 | 0.045 | 0.000 | 0.000 | 0.010 |
|  | **285** | 0.011 | 0.010 | 0.000 | 0.009 | 0.000 | 0.000 | 0.010 |
|  | **287** | 0.011 | 0.000 | 0.000 | 0.009 | 0.017 | 0.000 | 0.010 |
|  | **291** | 0.000 | 0.000 | 0.000 | 0.009 | 0.000 | 0.000 | 0.000 |
|  | **297** | 0.000 | 0.000 | 0.000 | 0.009 | 0.008 | 0.000 | 0.000 |
|  | **299** | 0.000 | 0.000 | 0.000 | 0.009 | 0.000 | 0.000 | 0.000 |
|  | ***Ho*** | 0.523 | 0.840 | 0.771 | 0.455 | 0.433 | 0.745 | 0.625 |
|  | ***He*** | 0.798 | 0.771 | 0.733 | 0.812 | 0.678 | 0.679 | 0.779 |
|  | ***F*** | 0.348 | -0.090 | -0.052 | 0.442 | 0.363 | -0.099 | 0.200 |
|  | **p of HWE** | 0.000 | 0.951 | 0.424 | 0.000 | 0.000 | 0.997 | 0.126 |
|  |  |  |  |  |  |  |  |  |
| **D3S1285** | **233** | 0.000 | 0.000 | 0.000 | 0.000 | 0.000 | 0.000 | 0.010 |
|  | **237** | 0.022 | 0.040 | 0.031 | 0.018 | 0.017 | 0.049 | 0.082 |
|  | **239** | 0.089 | 0.070 | 0.229 | 0.145 | 0.333 | 0.196 | 0.286 |
|  | **241** | 0.211 | 0.220 | 0.167 | 0.082 | 0.250 | 0.235 | 0.173 |
|  | **243** | 0.522 | 0.510 | 0.427 | 0.573 | 0.233 | 0.314 | 0.235 |
|  | **245** | 0.156 | 0.160 | 0.135 | 0.127 | 0.158 | 0.206 | 0.173 |
|  | **247** | 0.000 | 0.000 | 0.010 | 0.009 | 0.000 | 0.000 | 0.041 |
|  | **249** | 0.000 | 0.000 | 0.000 | 0.000 | 0.008 | 0.000 | 0.000 |
|  | **251** | 0.000 | 0.000 | 0.000 | 0.027 | 0.000 | 0.000 | 0.000 |
|  | **253** | 0.000 | 0.000 | 0.000 | 0.018 | 0.000 | 0.000 | 0.000 |
|  | ***Ho*** | 0.600 | 0.660 | 0.688 | 0.382 | 0.750 | 0.765 | 0.878 |
|  | ***He*** | 0.657 | 0.666 | 0.725 | 0.632 | 0.753 | 0.771 | 0.803 |
|  | ***F*** | 0.088 | 0.009 | 0.053 | 0.398 | 0.004 | 0.008 | -0.094 |
|  | **p of HWE** | 0.184 | 0.410 | 0.259 | 0.000 | 0.437 | 0.734 | 0.186 |
|  |  |  |  |  |  |  |  |  |
| **D3S1292** | **117** | 0.000 | 0.000 | 0.000 | 0.009 | 0.000 | 0.000 | 0.000 |
|  | **121** | 0.111 | 0.180 | 0.167 | 0.136 | 0.158 | 0.118 | 0.092 |
|  | **123** | 0.000 | 0.010 | 0.000 | 0.027 | 0.000 | 0.000 | 0.010 |
|  | **125** | 0.111 | 0.070 | 0.094 | 0.027 | 0.092 | 0.137 | 0.041 |
|  | **127** | 0.044 | 0.050 | 0.010 | 0.018 | 0.075 | 0.000 | 0.092 |
|  | **129** | 0.100 | 0.100 | 0.083 | 0.127 | 0.133 | 0.078 | 0.061 |
|  | **131** | 0.044 | 0.180 | 0.052 | 0.027 | 0.058 | 0.216 | 0.071 |
|  | **133** | 0.233 | 0.150 | 0.115 | 0.245 | 0.175 | 0.137 | 0.255 |
|  | **135** | 0.011 | 0.050 | 0.031 | 0.009 | 0.008 | 0.049 | 0.051 |
|  | **137** | 0.056 | 0.000 | 0.177 | 0.100 | 0.075 | 0.000 | 0.051 |
|  | **139** | 0.011 | 0.130 | 0.042 | 0.000 | 0.008 | 0.216 | 0.031 |
|  | **141** | 0.133 | 0.060 | 0.156 | 0.173 | 0.133 | 0.010 | 0.173 |
|  | **143** | 0.100 | 0.020 | 0.042 | 0.018 | 0.042 | 0.039 | 0.041 |
|  | **145** | 0.044 | 0.000 | 0.031 | 0.082 | 0.025 | 0.000 | 0.031 |
|  | **147** | 0.000 | 0.000 | 0.000 | 0.000 | 0.008 | 0.000 | 0.000 |
|  | **149** | 0.000 | 0.000 | 0.000 | 0.000 | 0.008 | 0.000 | 0.000 |
|  | ***Ho*** | 0.844 | 0.860 | 0.875 | 0.836 | 0.917 | 0.863 | 0.837 |
|  | ***He*** | 0.884 | 0.881 | 0.889 | 0.863 | 0.890 | 0.854 | 0.878 |
|  | ***F*** | 0.045 | 0.024 | 0.015 | 0.031 | -0.030 | -0.011 | 0.047 |
|  | **p of HWE** | 0.778 | 0.720 | 0.358 | 0.009 | 0.511 | 0.922 | 0.002 |
|  |  |  |  |  |  |  |  |  |
| **D3S1297** | **349** | 0.167 | 0.000 | 0.000 | 0.009 | 0.000 | 0.000 | 0.000 |
|  | **351** | 0.278 | 0.010 | 0.104 | 0.400 | 0.383 | 0.431 | 0.510 |
|  | **353** | 0.000 | 0.270 | 0.281 | 0.009 | 0.033 | 0.029 | 0.031 |
|  | **355** | 0.056 | 0.040 | 0.000 | 0.009 | 0.058 | 0.000 | 0.000 |
|  | **357** | 0.200 | 0.200 | 0.177 | 0.055 | 0.108 | 0.206 | 0.133 |
|  | **359** | 0.022 | 0.050 | 0.042 | 0.036 | 0.050 | 0.069 | 0.051 |
|  | **361** | 0.233 | 0.330 | 0.292 | 0.245 | 0.208 | 0.225 | 0.133 |
|  | **363** | 0.044 | 0.100 | 0.104 | 0.218 | 0.125 | 0.039 | 0.143 |
|  | **365** | 0.000 | 0.000 | 0.000 | 0.009 | 0.017 | 0.000 | 0.000 |
|  | **367** | 0.000 | 0.000 | 0.000 | 0.000 | 0.017 | 0.000 | 0.000 |
|  | **369** | 0.000 | 0.000 | 0.000 | 0.009 | 0.000 | 0.000 | 0.000 |
|  | ***Ho*** | 0.511 | 0.780 | 0.688 | 0.745 | 0.767 | 0.745 | 0.633 |
|  | ***He*** | 0.804 | 0.772 | 0.789 | 0.734 | 0.781 | 0.721 | 0.688 |
|  | ***F*** | 0.367 | -0.011 | 0.130 | -0.016 | 0.019 | -0.034 | 0.081 |
|  | **p of HWE** | 0.000 | 0.588 | 0.007 | 0.009 | 0.017 | 0.206 | 0.157 |
|  |  |  |  |  |  |  |  |  |
| **D3S1304** | **256** | 0.000 | 0.060 | 0.000 | 0.000 | 0.000 | 0.000 | 0.000 |
|  | **258** | 0.167 | 0.020 | 0.229 | 0.127 | 0.200 | 0.230 | 0.286 |
|  | **260** | 0.000 | 0.050 | 0.021 | 0.009 | 0.033 | 0.000 | 0.031 |
|  | **262** | 0.000 | 0.010 | 0.000 | 0.000 | 0.025 | 0.000 | 0.010 |
|  | **264** | 0.000 | 0.190 | 0.042 | 0.009 | 0.033 | 0.200 | 0.000 |
|  | **266** | 0.200 | 0.360 | 0.177 | 0.255 | 0.108 | 0.270 | 0.163 |
|  | **268** | 0.311 | 0.140 | 0.271 | 0.155 | 0.233 | 0.150 | 0.173 |
|  | **270** | 0.289 | 0.130 | 0.198 | 0.282 | 0.133 | 0.140 | 0.163 |
|  | **272** | 0.033 | 0.030 | 0.063 | 0.136 | 0.083 | 0.010 | 0.092 |
|  | **274** | 0.000 | 0.010 | 0.000 | 0.027 | 0.133 | 0.000 | 0.082 |
|  | **276** | 0.000 | 0.000 | 0.000 | 0.000 | 0.017 | 0.000 | 0.000 |
|  | ***Ho*** | 0.867 | 0.800 | 0.813 | 0.618 | 0.733 | 0.740 | 0.816 |
|  | ***He*** | 0.759 | 0.798 | 0.806 | 0.804 | 0.855 | 0.800 | 0.827 |
|  | ***F*** | -0.143 | -0.002 | -0.008 | 0.232 | 0.144 | 0.076 | 0.013 |
|  | **p of HWE** | 0.206 | 0.563 | 0.848 | 0.016 | 0.088 | 0.002 | 0.497 |
|  |  |  |  |  |  |  |  |  |
| **D3S1580** | **219** | 0.033 | 0.000 | 0.000 | 0.000 | 0.017 | 0.000 | 0.000 |
|  | **221** | 0.022 | 0.050 | 0.000 | 0.009 | 0.000 | 0.000 | 0.000 |
|  | **223** | 0.122 | 0.050 | 0.094 | 0.009 | 0.067 | 0.049 | 0.000 |
|  | **225** | 0.022 | 0.010 | 0.021 | 0.009 | 0.042 | 0.029 | 0.010 |
|  | **227** | 0.089 | 0.060 | 0.167 | 0.155 | 0.142 | 0.167 | 0.173 |
|  | **229** | 0.033 | 0.070 | 0.010 | 0.055 | 0.025 | 0.020 | 0.041 |
|  | **231** | 0.033 | 0.100 | 0.104 | 0.027 | 0.067 | 0.010 | 0.122 |
|  | **233** | 0.078 | 0.230 | 0.177 | 0.227 | 0.267 | 0.186 | 0.102 |
|  | **235** | 0.278 | 0.260 | 0.292 | 0.373 | 0.167 | 0.343 | 0.245 |
|  | **237** | 0.200 | 0.120 | 0.115 | 0.100 | 0.200 | 0.127 | 0.153 |
|  | **239** | 0.033 | 0.020 | 0.021 | 0.009 | 0.008 | 0.029 | 0.133 |
|  | **241** | 0.000 | 0.020 | 0.000 | 0.000 | 0.000 | 0.010 | 0.000 |
|  | **243** | 0.033 | 0.000 | 0.000 | 0.009 | 0.000 | 0.020 | 0.020 |
|  | **245** | 0.011 | 0.000 | 0.000 | 0.000 | 0.000 | 0.010 | 0.000 |
|  | **247** | 0.011 | 0.000 | 0.000 | 0.018 | 0.000 | 0.000 | 0.000 |
|  | **251** | 0.000 | 0.010 | 0.000 | 0.000 | 0.000 | 0.000 | 0.000 |
|  | ***Ho*** | 0.867 | 0.980 | 0.854 | 0.673 | 0.833 | 0.804 | 0.837 |
|  | ***He*** | 0.857 | 0.849 | 0.831 | 0.778 | 0.836 | 0.806 | 0.850 |
|  | ***F*** | -0.012 | -0.156 | -0.029 | 0.137 | 0.004 | 0.003 | 0.016 |
|  | **p of HWE** | 0.732 | 0.053 | 0.064 | 0.011 | 0.392 | 0.000 | 0.447 |
|  |  |  |  |  |  |  |  |  |
| **D3S1614** | **105** | 0.011 | 0.000 | 0.000 | 0.000 | 0.050 | 0.010 | 0.010 |
|  | **107** | 0.011 | 0.110 | 0.125 | 0.173 | 0.050 | 0.088 | 0.020 |
|  | **109** | 0.478 | 0.410 | 0.438 | 0.427 | 0.392 | 0.392 | 0.429 |
|  | **111** | 0.022 | 0.040 | 0.031 | 0.064 | 0.017 | 0.010 | 0.071 |
|  | **113** | 0.100 | 0.180 | 0.083 | 0.036 | 0.092 | 0.147 | 0.204 |
|  | **115** | 0.044 | 0.040 | 0.073 | 0.055 | 0.100 | 0.059 | 0.082 |
|  | **117** | 0.033 | 0.030 | 0.021 | 0.009 | 0.050 | 0.010 | 0.031 |
|  | **119** | 0.300 | 0.170 | 0.219 | 0.209 | 0.192 | 0.137 | 0.143 |
|  | **121** | 0.000 | 0.020 | 0.010 | 0.018 | 0.058 | 0.118 | 0.010 |
|  | **123** | 0.000 | 0.000 | 0.000 | 0.009 | 0.000 | 0.029 | 0.000 |
|  | ***Ho*** | 0.733 | 0.760 | 0.792 | 0.691 | 0.717 | 0.863 | 0.755 |
|  | ***He*** | 0.675 | 0.762 | 0.739 | 0.742 | 0.787 | 0.787 | 0.749 |
|  | ***F*** | -0.087 | 0.002 | -0.072 | 0.069 | 0.090 | -0.097 | -0.009 |
|  | **p of HWE** | 0.595 | 0.766 | 0.641 | 0.156 | 0.366 | 0.724 | 0.947 |

n: number of chromosomes;

*: genotyped in this paper.
