# Supplementary material for: The Adaptive Change of HLA-DRB1 Allele Frequencies Caused by Natural Selection in a Mongolian Population That Migrated to the South of China
Source: PLoS One. 2015 Jul 31;10(7):e0134334. doi: 10.1371/journal.pone.0134334 (PMC4521750; doi:10.1371/journal.pone.0134334)
Supplement: S3 Table — (DOC) [file pone.0134334.s005.doc]

**Table S3. Likelihood values and parameter estimates for HLA-DRB1 exon 2 in the five** populations

| **Pop** | **Model code** | **l** | **Average *d*_N_/*d*_S_** | **Parameter estimates*** |
| --- | --- | --- | --- | --- |
| Han | **M1a**(nearly neutral) | -1104.2 | 0.294 | *p*0 = 0.721, *p*1 = 0.279  *ω*0 = 0.020, *ω*1 = 1.000 |
|  | **M2a**(positive selection) | -1085.0 | 0.750 | *p*0 = 0.690, *p*1 = 0.209, *p*2 = 0.101  *ω*0 = 0.022, *ω*1 = 1.000, *ω*2 = 5.219 |
|  | **M7**(beta) | -1106.0 | 0.219 | a = 0.014, b = 0.046 |
|  | **M8**(beta&w>1) | -1085.8 | 0.721 | *p*0 = 0.897, *p*2 = 0.103  a = 0.0187, b = 0.061 *ω*2 = 5.081 |
| Hani | **M1a**(nearly neutral) | -826.0 | 0.317 | *p*0 = 0.692, *p*1 = 0.308  *ω*0 = 0.014, *p*1 = 1.000 |
|  | **M2a**(positive selection) | -807.7 | 0.71 | *p*0 = 0.659, *p*1 = 0.317, *p*2 = 0.024  *ω*0 = 0.006, *ω*1 = 1.000, *ω*2 = 15.903 |
|  | **M7**(beta) | -826.0 | 0.314 | a = 0.014, b = 0.028 |
|  | **M8**(beta&w>1) | -807.8 | 0.685 | *p*0 = 0.975, *p*2 = 0.025  a = 0.011, b = 0.023, *ω*2 = 15.623 |
| Dai | **M1a**(nearly neutral) | -1251.9 | 0.293 | *p*0 = 0.721, *p*1 = 0.279  *ω*0 = 0.019, *ω*2 = 1.000 |
|  | **M2a**(positive selection) | -1229.2 | 0.634 | *p*0 = 0.698, *p*1 = 0.234, *p*2 = 0.068  *ω*0 = 0.020, *ω*1 = 1.000, *p*2 = 5.697 |
|  | **M7**(beta) | -1251.4 | 0.315 | a = 0.012, b = 0.024 |
|  | **M8**(beta&w>1) | -1230.5 | 0.586 | *p*0 = 0.929, *p*2 = 0.071  a = 0.017, b = 0.055, *ω*2 = 5.402 |
| Yao | **M1a**(nearly neutral) | -1040.6 | 0.332 | *p*0 = 0.678, *p*1 = 0.322  *ω*0 = 0.015, *ω*2 = 1.000 |
|  | **M2a**(positive selection) | -1018.3 | 0.880 | *p*0 = 0.635, *p*1 = 0.247, *p*2 = 0.118  *ω*0 = 0.012, *ω*1 = 1.000, *ω*2 = 5.289 |
|  | **M7**(beta) | -1040.7 | 0.318 | a = 0.013, b = 0.025 |
|  | **M8**(beta&w>1) | -1018.3 | 0.907 | *p*0 = 0.882, *p*2 = 0.118  a = 0.013, q = 0.026, *ω*2 = 5.415 |
| Wa | **M1a**(nearly neutral) | -993.3 | 0.291 | *p*0 = 0.725, *p*1 = 0.275  *ω*0 = 0.022, *ω*1 = 1.000 |
|  | **M2a**(positive selection) | -970.3 | 0.840 | *p*0 = 0.698, *p*1 = 0.252, *p*2 = 0.050  *ω*0 = 0.023, *ω*1 = 1.000, *ω*2 = 11.424 |
|  | **M7**(beta) | -994.6 | 0.315 | a = 0.013, b = 0.027 |
|  | **M8**(beta&w>1) | -971.1 | 0.900 | *p*0 = 0.949, *p*2 = 0.051  a = 0.014, b = 0.029, *ω*2 = 11.889 |

**ω*0 is the ratio ofnonsynonymous-synonymous substitutions (*d*_N_/*d*_S_) of sites, with the proportion of *p*0, at which nonsynonymous mutations are “slightly deleterious”; *ω*1 is the *d*_N_/*d*_S_ of completely neutral sites (*ω*1 = 1) with a proportion of *p*1; *ω*2 is the *d*_N_/*d*_S_ of positively selected sites with a proportion of *p*2. The ‘a’ and ‘b’ are the shape parameters of the beta distribution.
