# Supplementary material for: The Adaptive Change of HLA-DRB1 Allele Frequencies Caused by Natural Selection in a Mongolian Population That Migrated to the South of China
Source: PLoS One. 2015 Jul 31;10(7):e0134334. doi: 10.1371/journal.pone.0134334 (PMC4521750; doi:10.1371/journal.pone.0134334)
Supplement: S4 Table — (DOC) [file pone.0134334.s006.doc]

**Table S4. Population pairwise *F*_ST_** values based on STR analysis

| **Pop** | **Han** | **Mongolian_IM** | **Mongolian_YN** | **Hani** | **Dai** | **Yao** | **Wa** |
| --- | --- | --- | --- | --- | --- | --- | --- |
| **Han** | **-** | **0.000** | **0.000** | **0.000** | **0.000** | **0.000** | **0.000** |
| **Mongolian_IM** | 0.026 | **-** | **0.000** | **0.000** | **0.000** | **0.000** | **0.000** |
| **Mongolian_YN** | 0.013 | 0.015 | **-** | **0.000** | **0.000** | **0.000** | **0.000** |
| **Hani** | 0.014 | 0.034 | 0.019 | **-** | **0.000** | **0.000** | **0.000** |
| **Dai** | 0.023 | 0.039 | 0.018 | 0.022 | **-** | **0.000** | **0.000** |
| **Yao** | 0.031 | 0.029 | 0.027 | 0.031 | 0.019 | **-** | **0.000** |
| **Wa** | 0.021 | 0.045 | 0.026 | 0.019 | 0.009 | 0.022 | **-** |

FST values are shown in the lower triangle, and the P values are shown in the upper triangle. P values less than 0.002 (after Bonferroni correction) are in bold.
