# Supplementary material for: The Adaptive Change of HLA-DRB1 Allele Frequencies Caused by Natural Selection in a Mongolian Population That Migrated to the South of China
Source: PLoS One. 2015 Jul 31;10(7):e0134334. doi: 10.1371/journal.pone.0134334 (PMC4521750; doi:10.1371/journal.pone.0134334)
Supplement: S5 Table — (DOC) [file pone.0134334.s007.doc]

**Table S5. Population pairwise *F*_ST_ values based on alleles frequencies of HLA-DRB1**

| **Pop** | **Han** | **Mongolian_IM** | **Mongolian_YN** | **Hani** | **Dai** | **Yao** | **Wa** |
| --- | --- | --- | --- | --- | --- | --- | --- |
| **Han** | **-** | 0.801 | **0.000** | **0.000** | **0.000** | **0.000** | **0.000** |
| **Mongolian_IM** | 0.000 | **-** | **0.000** | **0.000** | **0.000** | **0.000** | **0.000** |
| **Mongolian_YN** | 0.045 | 0.054 | **-** | **0.000** | **0.000** | **0.000** | 0.007 |
| **Hani** | 0.073 | 0.071 | 0.061 | **-** | **0.000** | **0.000** | **0.000** |
| **Dai** | 0.027 | 0.029 | 0.033 | 0.039 | **-** | **0.000** | **0.000** |
| **Yao** | 0.039 | 0.032 | 0.074 | 0.030 | 0.025 | **-** | **0.000** |
| **Wa** | 0.080 | 0.093 | 0.011 | 0.088 | 0.055 | 0.120 | **-** |

FST values are shown in the lower triangle, and P values are shown in the upper triangle. P values less than 0.002 (after Bonferroni correction) are in bold.
